# Online Trajectory Estimation Based on a Network-Wide Cellular Fingerprint Map

**DOI:** 10.3390/s22041605

**Published:** 2022-02-18

**Authors:** Langqiao Chen, Yuhuan Lu, Zhaocheng He, Yixian Chen

**Affiliations:** 1 School of Intelligent Systems Engineering, Sun Yat-sen University, Guangzhou 510275, China; chenlq8@mail2.sysu.edu.cn (L.C.); chenyx96@mail2.sysu.edu.cn (Y.C.); 2 Department of Computer and Information Science, University of Macau, Taipa, Macao 999078, China; yc17462@connect.um.edu.mo; 3 State Key Laboratory of Internet of Things for Smart City, University of Macau, Taipa, Macao 999078, China

**Keywords:** intelligent transportation systems, traffic monitoring, human mobility, data analysis

## Abstract

Cellular signaling data is widely available in mobile communications and contains abundant movement sensing information of individual travelers. Using cellular signaling data to estimate the trajectories of mobile users can benefit many location-based applications, including infectious disease tracing and screening, network flow sensing, traffic scheduling, etc. However, conventional methods rely too much on heuristic hypotheses or hardware-dependent network fingerprinting approaches. To address the above issues, NF-Track (Network-wide Fingerprinting based Tracking) is proposed to realize accurate online map-matching of cellular location sequences. In particular, neither prior assumptions such as arterial preference and less-turn preference or extra hardware-relevant parameters such as RSS and SNR are required for the proposed framework. Therefore, it has a strong generalization ability to be flexibly deployed in the cloud computing environment of telecom operators. In this architecture, a novel segment-granularity fingerprint map is put forward to provide sufficient prior knowledge. Then, a real-time trajectory estimation process is developed for precise positioning and tracking. In our experiments implemented on the urban road network, NF-Track can achieve a recall rate of 91.68% and a precision rate of 90.35% in sophisticated traffic scenes, which are superior to the state-of-the-art model-based unsupervised learning approaches.

## 1. Introduction

Mobile phones have become a kind of universal instant messengers nowadays, and their users generate huge amounts of real-time records that form the backbone of ubiquitous computing in heterogeneous sensor networks [1,2,3]. As the most well-applied one, GPS positioning data has given vital information for many applications, including traffic sensing, traffic incident detection, travel prediction, route recommendations, etc. [4,5,6] However, mobile users such as regular commuters would prefer to keep GPS off while unnecessary. Hence, the backend systems of GPS service providers could only observe partial location data of users. As a supplement to GPS localization, cellular signaling data has become a promising type of mobile phone data that ceaselessly records the signaling between base stations and every mobile device. It has been widely used in location-based services, including route recommendation, traffic scheduling, etc. Thus, a large number of technologies have been proposed for user location sensing based on cellular data [7,8,9,10,11].

As an extensive application originating from user localization, real-time and accurate estimation of a cellular-based trajectory is of great significance for urban individual movement monitoring and management nowadays (i.e., infectious disease tracing and screening [12,13,14] and network flow sensing and controlling [15,16,17,18,19,20]), because cellular signaling data can be passively sent back to the cloud systems in real time and contains a wealth of movement information of individual travelers. An online trajectory estimation based on cellular signaling sequences has been widely investigated for years. Some studies have attempted to filter large localization errors and directly decouple the movement trajectory under network constraints [21,22,23,24]. However, cleaned cellular localization was still unsatisfactory in dense urban road networks, because the positioning errors might probably be larger than the road span. Some other researchers innovatively applied advanced deep learning approaches in the trajectory estimation process and achieved good performance in their field test [25]. Nonetheless, the deep learning-based models need large amounts of ground truth trajectories for convergence, and therefore, their utility is limited. For these reasons, the mainstream of the existing solutions was based on the Hidden Markov Model (HMM) framework, which could combine the interfering noise and map-matching rules into the trajectory to reinforce the inference process [26,27,28].

Most of the above methods are characterized by unsupervised learning and require a certain amount of prior knowledge to accurately track a mobile device. An enormous number of manual conceptions and rules, including arterial preference, less-turn preference, and same direction preference, were predefined in such unsupervised learning approaches, which weakens the generalization ability of the models, because travelers will not always behave according to these predefinitions due to their various transport modes and dynamic route choices. Hence, how to free the estimation process from these prior assumptions becomes a meaningful question to be answered.

Nowadays, fingerprint mapping is utilized as the mainstream localization by associating the locations in the testing field with a unique wireless signal feature [29,30,31,32]. On this basis, a mobile device can match itself to a specific location with the real-time measured signal features. Therefore, a fingerprint map is capable of providing sufficient prior knowledge, and many previous studies endeavored to migrate it to road network scenarios for precise positioning and tracking [33,34,35,36]. However, the majority of location fingerprinting methods are hardware-dependent, because the construction fingerprint map and positioning process require the RSS (Received Signal Strength), SNR (Signal-to-Noise Ratio) and TA (Time Advanced) information, which is only available with special devices and violates the ubiquity [24,37]. For this reason, these approaches are not lightweight enough to be applied to the cloud computing backend of telecom operators with RSS absence, which means conventional fingerprinting methods can only locate a mobile phone itself actively rather than calculate at the network side passively on a large scale. Therefore, conventional fingerprinting methods need some promotion to overcome these restrictions. In this paper, we revisit the map-matching task from the data perspective and propose to utilize the great power of data to help solve the aforementioned problems that obstruct conventional unsupervised methods. Therefore, a novel online trajectory estimation framework, NF-Track (Network-wide Fingerprinting based Tracking), is presented. Unlike the current fingerprinting technologies for mobile device localization that calibrate many extra hardware-relevant parameters at the grid scale, NF-Track provides more accurate localization by dividing the area of interest into road segments, and the fingerprint features are constructed with road segments as the basic units. This innovation not only avoids the trouble of standing at each grid for a certain time to draw the signal strength histogram but also benefits the trajectory estimation, as the mobile device is mostly on the road network. On the basis of the built segment-granularity fingerprinting map, an anchor-based similarity calculation model is developed to achieve online prediction. Compared to unsupervised methods, the proposed online prediction architecture depends on prior knowledge about segment-level signal features given by fingerprint map, which enhances the stability and precision of trajectory estimation.

To sum up, the major innovations of NF-Track are as follows:

(1) Distinct from current fingerprinting technologies that are hardware-dependent, NF-Track is supported by a data-driven fingerprint map, which not only improves the efficiency of signal collection but also benefits online cellular location sequence map-matching.
(2) On the basis of such fingerprint map, the proposed trajectory estimation algorithm is independent of either hardware-relevant information in conventional fingerprinting approaches or heuristic hypotheses that are widely leveraged by unsupervised methods. Therefore, NF-Track is suitable for being deployed over cloud computing backends where only cellular localization is available.
(3) We conduct our experiments on a real-world urban dataset. The results demonstrated the significant advantages of our real-time trajectory estimation approach contrasted with the current state-of-the-art online map-matching algorithm, especially for the estimation of irregular trajectories that are more twisted than the regular trajectories that prefer the shortest and straightest paths.

The rest of this paper is organized as follows. In Section 2, we discuss the relevant literature. In Section 3, we elaborate on the data preprocessing and the problem formulation. Section 4 details the design of our NF-Track. Section 5 presents the performance evaluation of our framework. Finally, Section 6 concludes this paper.

## 2. Literature Review

### 2.1. Location Fingerprinting-Based Positioning

**Figure 1:**
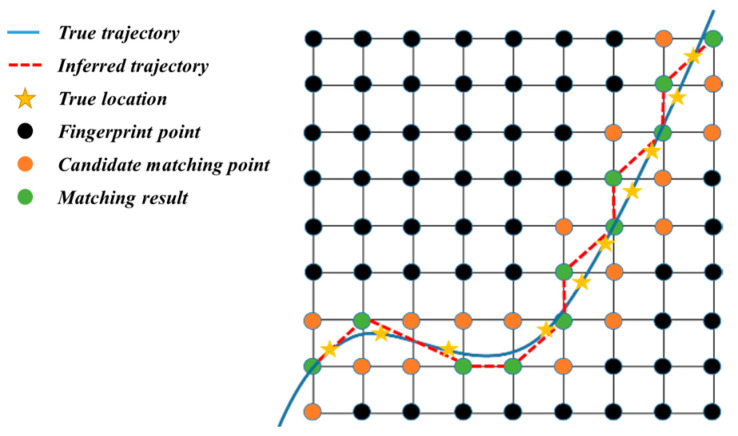
The schematic of wireless device positioning and tracking based on the indoor fingerprint map.

Wireless device positioning and tracking are of great significance for individual movement monitoring and management in sensor networks. Due to the exclusion of GPS signals in indoor areas, location fingerprinting is proposed to determine devices’ locations in wireless areas based on the measured signal features, e.g., Received Signal Strength (RSS), Signal-to-Noise Ratio (SNR) and Time Advanced (TA) [29,30,31,32]. Specifically, location fingerprinting is the process of associating locations in an environment with some sort of wireless signal feature that is unique to that location. The location fingerprinting-based positioning methods can be divided into two stages [38]. One is the offline fingerprint map construction, and the other is the online location estimation. In the offline stage, a rectangular grid of points is cast on the two-dimensional space, as Figure 1 shows below. Each spatial point is calibrated with a basic fingerprint that is associated with a unique feature constructed from the signal propagation characteristics. After all points are calibrated and stored, the fingerprint map is constructed. Then, in the online stage, while the signal features like RSS and SNR are measured by the device online, we can infer its location to a spatial point whose fingerprint is most similar to the measurement results. If the signal features are obtained continuously, the device can be tracked by connecting its estimated locations.

Some research migrated the location fingerprinting-based positioning methods to urban environments later, where the location in communication space is associated with a unique fingerprint structured by the signal features observed from the surrounding cellular base stations, and then, the outdoor mobile device can be located online. For example, to cope with the absence of GPS positioning under some circumstances, Ibrahim and Youssef [8] built an RSS-based location fingerprinting system for mobile phone positioning in the area of interest. Chen et al. [39] made a great effort to create a cellular location fingerprinting system in a metropolitan environment with higher base station density and achieved good performance in the mobile device position estimation.

Besides the outdoor area, some researchers endeavored to apply fingerprinting location technology to road networks, because the localization of mobile devices along a route is meaningful to many traffic management applications like the travel time estimation [31]. They firstly divided the road into several segments, and then, the distribution of the RSS from different base stations was measured as a segment-associated fingerprint for each segment. From then on, mobile devices’ segment-granularity positioning could be realized by observing their RSS distribution online.

These methods provided a new perspective for individual positioning and tracking based on the prior knowledge extracted from observed features. It can be seen that location fingerprinting technologies have great potential in urban traveler management.

### 2.2. Online Trajectory Estimation

The online trajectory estimation is the task of mapping a set of real-time locations with errors to the corresponding points on the road network. The location data comes from a variety of moving objects, such as vehicles and mobile phones. In the current transportation research, GPS data is the spatiotemporal locations that are most commonly used. Online trajectory estimation technologies were early applied to align the low-sampling-rate GPS series to the digital road network [40,41,42]. The general processing of online trajectory is based on sliding window methods, which divide the trajectory into several input sequences and handle them independently. The window size is the significant parameter that affects overall performance. In previous studies, many efforts were made to investigate the estimation accuracies and computing latencies in different window sizes [42,43]. To promote the system performance, researchers proposed various sliding window algorithms, including fixed sliding window, bounded variable sliding window, adjustable sliding window, etc. [4,42,44]. These works benefit many real-time continuous location-based services.

Besides GPS data, cellular data contains large-scale spatiotemporal movement information of urban travelers because mobile phones have a very high penetration rate among citizens nowadays. Therefore, how to estimate their trajectories with cellular signaling sequences becomes the subject of growing concern.

### 2.3. Cellular Signaling Sequence Map-Matching

Due to the sparseness and fuzziness of cellular data, cellular signaling sequence map-matching are mainly targets at addressing the issues of uncertainty. Some researchers tried to decouple individual movement information from a cellular signaling sequence by means of clustering, interpolation, etc. to realize the trajectory reconstruction on road networks [21,22,23,24]. These methods can directly clean and smooth the cellular signaling sequences, quickly analyze the staying and moving behavior of travelers and extract their approximate spatial trajectories. Herein, density-based clustering algorithms are mainly used to partition cellular localizations into several groups, from which low-density localizations can be obtained. Meanwhile, many studies made efforts to promote the clustering performance by further investigating the intrinsic clustering structure [45,46], features employment [47] and noise robustness [48]. These efforts were meant to capture higher accuracy localizations of travelers. However, due to the complexity of road network, it might lead to a situation where there were many reasonable candidate matching paths for a certain cellular signaling sequence. Thus, many of these methods could only approximate individual cellular signaling sequence to the main traffic corridors of the city rather than a specific path, which was limited to implement refined management and guidance on travelers.

Therefore, the individual movement information extracted from the cellular signaling sequence needs to be extensively reinforced to achieve accurate trajectory estimation. The fundamental way is to introduce prior rules to aid in fine-grained map-matching. Hence, many studies developed various rule-based methods to combine prior rules such as travelers’ path selection preferences in order to achieve a well-estimated individual trajectory. Yuan Y et al. [49] matched the cellular signaling sequence to the suburban road network well based on the assumption that drivers tended to choose high-grade roads. As for the urban environment, many studies used HMM algorithms to decode the cellular sequence to a mobile trajectory, which could take the noise of cellular data into account and enhance the map-matching performance [24,28]. However, these methods only performed in offline mode and did not deal with the live stream of the input locations, which limited the power of real-time positioning data.

Enlighted by existing online map-matching algorithms that used a sliding window to handle the input sequence incrementally [4,42], several HMM models were modified and introduced many heuristic mechanisms to the online trajectory inference process. Mohamed et al. [26] realized real-time trajectory estimation by an HMM that incorporated several heuristics like major road and shortest path preference to reduce the noise in the map-matching process. Due to travelers who would decide their route based on the estimation of traffic status and travel time on roads [50,51], Jagadeesh et al. [27] proposed an HMM-based map-matching framework with a pre-estimated route choice model, which was robust for inaccurate and sparse location data. Besides HMM-based methods, advanced deep learning approaches were brought in the application of online map-matching of cellular signaling sequences. For instance, Shen Z et al. [25] innovatively trained recurrent neural networks to capture the mobility pattern from enormous cellular data and then realized the trajectory estimation. Nonetheless, in order to ensure the convergence of the model, the deep-learning algorithm needed not only large amounts of sample trajectories for offline training but also many heuristic hypotheses of arterial preference, same direction preference and less-turn preference.

These online map-matching methods had the following two main limitations. One is that some of them could only achieve fine results in wide-range suburban areas or urban high-grade road networks whose topology was relatively simple; therefore, the heuristic assumptions mentioned above are compatible with them. However, that was not the case for crisscrossed urban road networks. The other is that such methods were highly dependent on prior assumptions to ensure the estimation accuracy, which would weaken the generalization ability of models. Therefore, it is worthwhile to realize the flexible tracking of free-moving and irregular travelers in urban environments purely based on cellular signaling data.

Therefore, different from the aforementioned unsupervised methods that rely too much on the heuristic hypothesis, some researchers attempted to develop cellular trajectory map-matching methods based on location fingerprinting technology, which would not rely on any assumptions about travelers’ preferences. For example, Thiagarajan et al. [34] divided the area of interest into uniform square grids and associated a set of observed base stations and their RSS values with each grid offline. The cellular signaling sequence was first matched to a sequence of traversed grids through HMM methods, and then, that grid sequence was further matched to road segments. Dalla et al. [35] observed the surrounding base stations and their signal strengths at the spatial points along the road, which were regarded as basic cellular fingerprints, and exploited in the trajectory estimation process.

The generalization of these fingerprint-based trajectory estimation methods was restricted from two perspectives. On the one hand, these approaches were hardware-relevant, because they relied on extra parameters like RSS and SNR. Hence, they were hard to be applied to the cloud computing systems of telecom operators whose signaling data contained cellular location only [37]. On the other hand, in order to build up a location fingerprinting system, these fingerprint-based trajectory estimation methods commonly associate the road network with massive observation points, and therefore, the fingerprinting process is quite inefficient based on point-grained fingerprint maps. Furthermore, point-grained location fingerprinting has another drawback that the concentration of fingerprint points might be hard to differentiate the signal features from each other and thereby increase the chance of positioning errors.

Inspired by this kind of research, we build a novel network-wide fingerprinting system for real-time cellular signaling sequences map-matching, which is well-behaved without either heuristic hypothesis or extra RSS information.

## 3. Preliminary

### 3.1. Data Acquisition and Preprocessing

**Figure 2:**
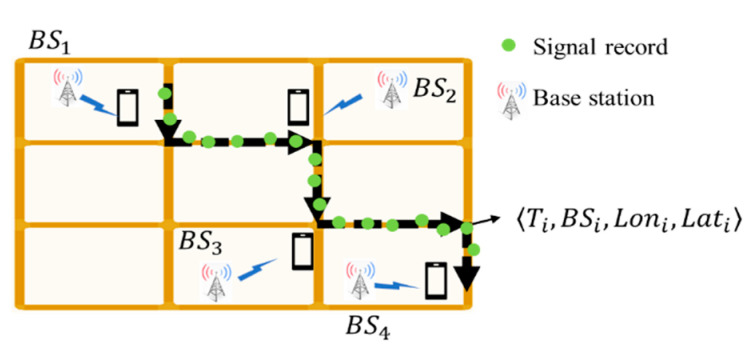
A schematical illustration of the data acquisition process.

Referring to previous network fingerprinting approaches [33,34,35], the benchmark data of this study is collected through war-driving on the road network, as Figure 2 illustrates.

Table 1 presents some record samples of the acquired dataset. A signaling record contains the Device ID, recorded timestamp and the connecting base station. Besides the cellular information, the GPS coordinates are recorded simultaneously (longitude and latitude) and regarded as the true location of the mobile device for modeling and validation. The frequency of data acquisition is around 1 Hz, which forms the benchmark dataset of this study.

### 3.2. Notation

The main notations in this paper are shown in Table 2.

### 3.3. Problem Formulation

A cellular signaling sequence obtained from telecom operators is a series of chronologically ordered locations generated by a mobile device, e.g., $\langle p_{1},p_{2},\;\ldots ,\;p_{k}\rangle$, where each location contains the geospatial coordinate of the signaling base station and a timestamp such as p=(lon, lat, t). The task of this study is transforming the cellular signaling sequence into a road segments series $\langle E_{1},\;E_{2},\;\ldots ,\;E_{n}\rangle$. As a result, the accuracy of the estimated trajectory can be evaluated by the ground truth GPS sequence.

## 4. Methodology

**Figure 3:**
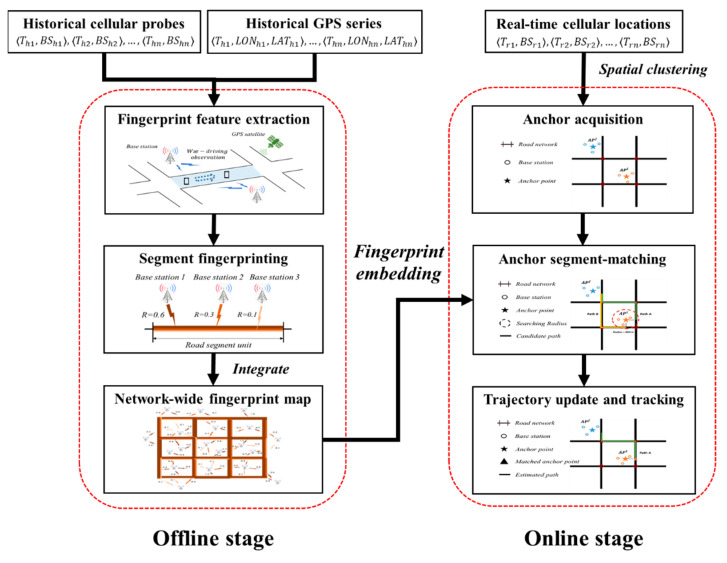
The two-stage trajectory estimation framework based on cellular signaling data.

In this section, we discuss the trajectory estimation architecture of NF-Track in detail. To be specific, the overview of the estimating framework (see Figure 3) is shown below.

Offline stage. In this stage, we first align the historical cellular series to the GPS series at the road segment scale. Then, the signal features of each road segment are extracted from the aligned cellular data as a unique fingerprint, and lastly, the obtained fingerprints constitute a network-wide cellular fingerprint map.

Online stage. For an input real-time cellular sequence, the online tracking process is implemented in the cloud computing backend of telecom operator as follows. Firstly, the spatial clustering method is applied to acquire a handful of high-confident anchors. Since each anchor is likely to have several candidate segments for map-matching, the preconstructed fingerprint map is taken as a reference to determine the most likely one. After that, the identified road segments are connected into a final trajectory.

### 4.1. Offline Stage

The fingerprint is a multidimensional feature vector extracted from the original cellular signal in essence [38]. Thus, our innovative segment-granularity fingerprint map is built by following steps.

#### 4.1.1. Fingerprint Feature Extraction

After network segmentation, we summarize the fingerprint features vector for each road segment. Actually, the observable information of cellular signaling data includes the user ID, signal recorded time and the connected base station. To characterize the features of cellular system in the road space based on these vanilla fields, the fingerprint features vector for a specific segment consists of two components: the set of stable impacting base stations and the corresponding set of impacting weights.

If a Stable impacting Base Station (SBS) has a stronger signal covering the current segment, mobile devices are more likely to connect with it subsequently. The grouped Stable impacting Base Station Sets (SBSSs) are probably distinct from one another due to the spatial heterogeneity of segments. Therefore, it is reasonable to consider SBSS as a fingerprint feature of the road segment. The extraction algorithm of SBSS is as follows.

Suppose n cellular sequences $\langle Seq_{1},\;Seq_{2},\;\ldots ,\;Seq_{n}\rangle$ are collected on the road segment E and m base stations $\langle BS_{1},\;BS_{2},\;\ldots ,\;BS_{m}\rangle$ have ever been recorded in them. Then, the frequency τ (1≤τ≤n) of each base station in the n cellular signaling sequences is counted. If τ>θ·n, the base station is designated as an SBS. Here, θ represents a critical threshold for SBS selection, and its value is set to 0.375 according to the sensitivity analysis in Section 5. Finally, all SBSs of E are put in a set, denoted by S(E).

It is insufficient to use S(E) as the fingerprint feature alone, because two road segments are possible to have the same fingerprint characteristics when they are very close to each other in space. Therefore, after the SBSs are sifted out, their impacting weights on the road segment are significant and further modeled. It is prone to recognizing that larger transmission power and less propagation loss give base stations a higher impact on the road segment. As for its residing mobile devices, their received signal strength is higher, and the connections are more stable. Therefore, the traveling mobile devices’ moving distance during connections with such base stations is probably longer.

Therefore, we propose the Cumulative Moving Distance (CMD) to indicate an SBS’s impacting weight on a road segment. For example, suppose there is a mobile device passing through a road segment E, and SBS is one of its S(E). Then, CMD(Seq, E, SBS) denotes the CMD of that mobile device while it communicates with SBS and moves on E, where Seq represents the generated cellular signaling sequence at that time. To eliminate the influence of randomness, the average CMD denoted by $\bar{\mathrm{CMD}}\left(E,\;SBS\right)$ is obtained from n collected cellular signaling sequences $\langle Seq_{1},Seq_{2},\;\ldots ,\;Seq_{n}\rangle$ as below to model the impacting weight of SBS on E.

(1) $$\bar{\mathrm{CMD}}\left(E,\;\mathrm{SBS}\right)=\frac{1}{k}\cdot \sum_{i=1}^{k}\mathrm{CMD}\left({\mathrm{Seq}}_{i},\;E,\;\mathrm{SBS}\right),$$

where $Seq_{i}$ represents the ith in k (1≤k≤n) cellular signaling sequences that contains the records of SBS. Furthermore, $\bar{\mathrm{CMD}}\left(E,\;SBS\right)$ needs to be normalized as R(E, SBS) shown below, because the lengths of the segments greatly affect their values.

Suppose segment E has m SBSs $\langle SBS_{1},SBS_{2},\;\ldots ,\;SBS_{m}\rangle$, and $R\left(E,\;SBS_{i}\right)$ (1≤i≤m) can be calculated as follows:

(2) $$R_{i}=R\left({E,\;\mathrm{SBS}}_{i}\right)=\frac{\bar{\mathrm{CMD}}\left({E,\;\mathrm{SBS}}_{i}\right)}{\sum_{t=1}^{m}\bar{\mathrm{CMD}}\left({E,\;\mathrm{SBS}}_{t}\right)},\;$$

which is regarded as $SBS_{i}$’s impacting weight on segment E.

Therefore, the weights of $\langle SBS_{1},SBS_{2},\;\ldots ,\;SBS_{m}\rangle$ on segment E can be represented by a one-dimensional vector $\left(R_{1},\;R_{2},\;\ldots ,\;R_{m}\right)$, which is

(3) $$R\left(E\right)=\left(R_{1}{,\;R}_{2}{,\;\ldots ,\;R}_{m}\right),$$

which satisfies $\sum_{i=1}^{m}R_{i}=1$. Herein, R(E) is defined as the fingerprint feature of segment E.

#### 4.1.2. Network-Wide Fingerprint Map Construction and Its Properties

After the fingerprint feature vectors of all road segments are calculated via previous steps, the network-wide fingerprint map can be constructed.

This section further illustrates that the fingerprint features of several segments can be combined to form the fingerprint of a path, which is the reference of cellular signaling sequence map-matching. Suppose a path P consists of a consecutive segment series $\langle E_{1},E_{2},\;\ldots ,\;E_{n}\rangle$. The SBSS of P is derived as follows.

(4) $$S\left(P\right)=S\left(E_{1},\;E_{2},\;\ldots ,\;E_{n}\right)=S\left(E_{1}\right)\;\cup \;S\left(E_{2}\right)\;\cup \;\ldots \;\cup \;S\left(E_{n}\right).$$

Suppose there are λ base stations in S(P), which is $S\left(P\right)=\left\{{\mathrm{SBS}}_{1}{,\;\mathrm{SBS}}_{2}{,\;\ldots ,\;\mathrm{SBS}}_{\lambda }\right\}$.

We have the R(P) representing the corresponding set of impacting weights of S(P), which is designated as the fingerprint feature of path P. The calculating procedure is as follows.

First, to facilitate the computing, $S\left(E_{i}\right)\;$and $R\left(E_{i}\right)$ are reset as $\;\tilde{S}\left(E_{i}\right)$ and $\;\tilde{R}\left(E_{i}\right)\;$for each $E_{i}$ in $\langle E_{1},E_{2},\;\ldots ,\;E_{n}\rangle$, where $\;\tilde{S}\left(E_{i}\right)=\left\{{\tilde{SBS}}_{i1},\;{\tilde{SBS}}_{i2},\;\cdots ,{\tilde{SBS}}_{i\lambda }\;\right\}\;$and $\tilde{R}\left(E_{i}\right)=\left[{\tilde{R}}_{i1},\;{\tilde{R}}_{i2},\;\ldots ,\;{\tilde{R}}_{i\lambda }\right]$.

Here,

(5) $${\tilde{SBS}}_{i\rho }=\{\begin{array}{c} SBS_{\rho },SBS_{\rho }\;\in \;S\left(E_{i}\right)\\ null,\;\;SBS_{\rho }\;\;\notin \;S\left(E_{i}\right) \end{array},$$

(6) $${\tilde{R}}_{i\rho }=\{\begin{array}{c} R_{i\pi }\;,SBS_{\rho }\;\in \;S\left(E_{i}\right)\\ \;\;0\;,\;\;SBS_{\rho }\;\notin \;S\left(E_{i}\right) \end{array},$$

where 1≤ρ≤λ, and π is the index of$\;SBS_{\rho }$ in $S\left(E_{i}\right)$.

Then, R(P) can be calculated by a weight vector α and an impacting weight matrix β as below:

(7) $$R\left(P\right){=\alpha }^{T}\beta ,$$

subject to

(8) $$\alpha ={\left[\theta_{1},\;\theta_{2},\;\ldots ,\theta_{n}\right]}^{T},$$

(9) $$\beta ={\left[\tilde{R}\left(E_{1}\right),\tilde{R}\left(E_{2}\right),\;\ldots ,\;\tilde{R}\left(E_{n}\right)\right]}^{T},$$

where $\theta_{i}$ stands for the weight of the ith segment in P, and $\sum_{i=1}^{n}\theta_{i}=1$. It can be calculated as follows:

(10) $$\theta_{i}=\;\frac{L(E_{i})}{\sum_{k=1}^{n}L(E_{k})\;},$$

where $L(E_{i})\;$denotes the length of the i th segment in $\langle E_{1},E_{2},\;\ldots ,\;E_{n}\rangle$.

In general, our fingerprint map has the following two highlights: (a) A novel segment-grained fingerprinting method based on the cellular signaling sequences is put forward to assist with mobile device positioning and city-wide trajectory estimation. (b) Two inventive features are created to replace the commonplace fingerprinting characteristics like RSS and SNR commonly leveraged by fingerprint maps. This improvement enables the real-time and accurate tracking of mobile devices in the cloud computing backends. On the basis of this fingerprint map, the base station handover sequences can be matched to the urban road network directly.

### 4.2. Online Stage

The online stage realizes the trajectory estimation in three steps: anchor clustering, anchor segment-matching and trajectory reconstruction. In the first step, all of the mobile device’s correlative base stations in a specific time span can be spatially clustered into several groups to decrease the positioning error resulted from cellular handover. The group centers with timestamps are denoted by Anchor Points (APs). The second step is to project all APs onto the road segments and generate the sub-trajectories between every two consecutive APs. In the final step, the real-time updated trajectory is produced by linking all the sub-trajectories.

#### 4.2.1. Step 1: Anchor Clustering

The signal of mobile devices is likely to switch back and forth between several neighboring base stations, which is known as the “Ping-Pong effect”, and brings interference to both spatial locating and trajectory estimation. Therefore, we adopt the DBSCAN (Density-based spatial clustering of applications with noise) algorithm [52] to incrementally generate spatially robust anchor points as signal sources using a time span of input cellular locations. In our model, the neighboring base stations are clustered according to the geodesic distances among them.

The DBSCAN algorithm has two main input parameters: MinPts and ε. Herein, MinPts is the minimum number of base stations to form a cluster, and the value is set as *2* to smooth the “Ping-Pong effect” as much as possible, while ε is the searching radius of a base station location. Notably, the distance between neighboring base stations is generally less than 300 m, which is consequently leveraged as the ε value.

For each clustering group with N base stations, the geometric center of its base stations’ positions is calculated by:

(11) $$\left(\mathrm{LON},\;\mathrm{LAT}\right)=\left(\frac{1}{N}\times \sum_{i=1}^{N}{\mathrm{BS}}_{i,\;\mathrm{LON}},\;\frac{1}{N}\times \sum_{i=1}^{N}{\mathrm{BS}}_{i,\;\mathrm{LAT}}\right).$$

Some base stations that cannot be classified into any clustering group are still set as anchor locations, because mobile devices also issue effective connections to them. Accordingly, the position of the that base station BS is exactly the anchor location.

(12) $$\left(\mathrm{LON},\;\mathrm{LAT}\right)=\left({\mathrm{BS}}_{\mathrm{LON}}{,\;\mathrm{BS}}_{\mathrm{LAT}}\right).\;$$

**Figure 4:**
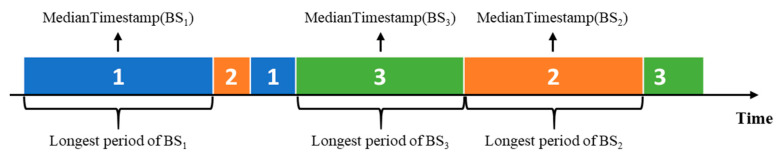
Timeline of the base stations handover in a cellular signaling sequence. The median timestamp of $BS_{i}$′ longest period is represented by a function $\mathrm{MedianTimestamp}\left(BS_{i}\right)$.

Furthermore, the timestamp of each anchor point is estimated, which paves the way for cellular signaling sequence map-matching in the following steps. We compute the timestamp for each anchor point, referring to the durations of the base stations lying in the corresponding group. For an instance shown in Figure 4, three base stations: $BS_{1}$, $BS_{2}$ and $BS_{3}$ are supposed to form an anchor point, whose timestamp T can be derived from the longest periods of $BS_{1}$, $BS_{2}$ and $BS_{3}$ as below:

(13) $$T=\frac{1}{N}\;\times \;\sum_{i=1}^{N}\mathrm{MedianTimestamp}\left({\mathrm{BS}}_{i}\right),$$

Here, N = 3 in this instance.

If the anchor point only corresponds to a base station BS, its timestamp T is given as follows:

(14) T=MedianTimestamp(BS).

The pseudo code of anchor point location capture and its timestamp inference are depicted by Algorithm 1.
**Algorithm 1.** Anchor point location capture and its timestamp inference.**Input:** Base station set obtained from a time span of input cellular locations 
          $\langle T_{1}{,\;\mathrm{BS}}_{1}\rangle {,\langle T}_{2}{,\;\mathrm{BS}}_{2}\rangle {,\ldots ,T}_{k}{,\;\mathrm{BS}}_{k}\to \left\{{\mathrm{BS}}_{1}{,\mathrm{BS}}_{2}{,\;\ldots ,\mathrm{BS}}_{n}\right\};$
Base stations—location mapping table.Distance function $\mathrm{Dist}\left(BS_{A},\;BS_{B}\right)$, which represents the distance between two base stations;Distance of neighborhood(ε);Minimum number of points(MinPts).
**Output**:
A series of chronologically ordered anchor points           $\mathrm{APS}=\left[\langle T_{1}{,\;\mathrm{AP}}_{1}\rangle {,\langle \;T}_{2}{,\;\mathrm{AP}}_{2}\rangle {,\;\ldots ,\;\langle T}_{m}{,\;\mathrm{AP}}_{m}\rangle \right].$
Implement DBSCAN algorithm: DBSCAN(BSS,Dist, ε, MinPts); Group the p clusters as a set $\left\{C_{1},\;C_{2},\;\ldots ,\;C_{p}\right\}$, and the q noise points as a set $\left\{NP_{1},\;NP_{2},\;\ldots ,\;NP_{q}\right\}$; Initiate a null set for APS; FOR each C IN $\left\{C_{1},\;C_{2},\;\ldots ,\;C_{p}\right\}$:   Take BSN the number of base stations in C;   $AP_{LON}=\frac{1}{BSN}\times \sum^{\;}BS_{LON}$, which is the average longitude of base stations in C;   $AP_{LAT}=\frac{1}{BSN}\times \sum^{\;}BS_{LAT}$; likewise,   $AP=\left(AP_{LON},AP_{LAT}\right)$;   Take T the inferred timestamp calculated by Equation(13);   APS+=〈T, AP〉; FOR each NP IN $\left\{NP_{1},\;NP_{2},\;\ldots ,\;NP_{q}\right\}$:   ${\mathrm{AP}}_{\mathrm{LON}}{=\mathrm{BS}}_{\mathrm{LON}}$, which is the longitude of the unique base station corresponding to NP;

#### 4.2.2. Step 2: Anchor Map Matching

**Figure 5:**
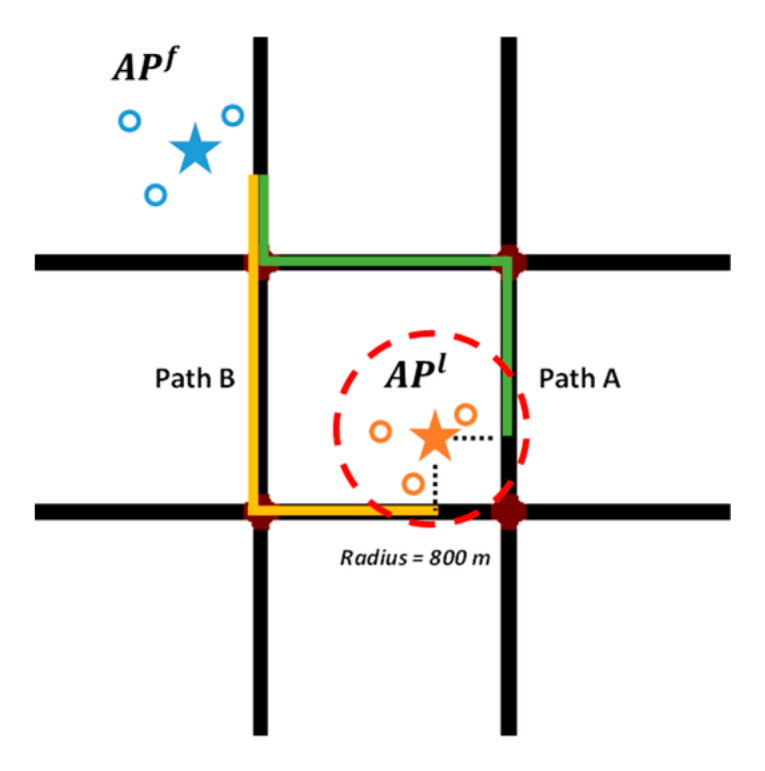
The orange anchor point probably has more than one matching option.

In the second step, we match the output anchor points onto the road network chronologically. The first one $AP_{1}$ is matched to its nearest road segment as origin, and the APs in the back is matched to specific road segments through fingerprinting process. As Figure 5 illustrates, $AP^{f}\;$and $AP^{l}$ are two consecutive APs. The former blue $AP^{f}$ has found its matching place, and the latter orange $AP^{l}$ is waiting to be matched. Due to its large signal coverage, $AP^{l}$ probably has more than one candidate segments. Therefore, from $AP^{f}$ to $AP^{l}$, there are two optional matching paths: $Path_{A}$ and $Path_{B}$. It should be noted that the AP’s searching radius directly affects the number of options, which is discussed in detail in Section 5.

Next, these two candidate paths are further investigated as below. By performing the following steps, the cellular signaling fragment between $AP^{f}\;$and $AP^{l}$ is intercepted and compared with the fingerprint features of these two candidate paths.

Firstly, we split out the target cellular signaling fragment $Seq_{infer}^{F}$ by the timestamps of $AP^{f}\;$and $AP^{l}$, which are denoted by $T_{infer}^{f}$ and $T_{infer,}^{l}$ respectively.

Suppose the input cellular signaling sequence is represented by $\langle T_{1},\;BS_{1}\rangle ,\langle T_{2},\;BS_{2}\rangle ,\ldots ,\langle T_{n},\;BS_{n}\rangle$ and the connecting base station at time $T_{infer}^{f}$ is $BS_{k}$ and that at $T_{infer}^{l}$ is $BS_{k+\alpha }$.

Thus, we have:

(15) $$Seq_{infer}^{F}=\left(T_{infer}^{f},\;BS_{k}\right),\;\left(T_{k+1},\;BS_{k+1}\right),\;\ldots ,\;\left(T_{infer}^{l},\;BS_{k+\alpha }\right),$$

satisfying $T_{k}\leq T_{infer}^{f}<T_{k+1}$ and $T_{k+\alpha -1}<T_{infer}^{l}\leq T_{k+\alpha }$.

Later, the recorded base stations set in $Seq_{infer}^{F}$ and their CMD proportion $R\left(Seq_{infer}^{F}\right)$ are extracted for the comparison with the fingerprint map in order to select the most likely candidate path.

The base stations can be extracted to a set $B\left({\mathrm{Seq}}_{\mathrm{infer}}^{F}\right)\;$as follows:

(16) $$B\left({\mathrm{Seq}}_{\mathrm{infer}}^{F}\right)\;=\left\{BS\;|\;BS\;exists\;in{\;\mathrm{Seq}}_{\mathrm{infer}}^{F}\right\}.$$

Suppose there are m base stations in $B\left({\mathrm{Seq}}_{\mathrm{infer}}^{F}\right)$; that is,

(17) $$B\left({\mathrm{Seq}}_{\mathrm{infer}}^{F}\right)=\left\{{\mathrm{BS}}_{1}{,\;\mathrm{BS}}_{2}{,\;\ldots ,\;\mathrm{BS}}_{m}\right\}.$$

The cumulative connection time of each base station in $B\left(Seq_{infer}^{F}\right)\;$is calculated, and they can be organized as a vector:

(18) $$W\left(Seq_{infer}^{F}\right)=\left(w_{1},\;w_{2},\;\ldots ,\;w_{m}\;\right).$$

Suppose the traveler moves at the velocity v, so $R\left(Seq_{infer}^{F}\right)$ can be evaluated by

(19) $$R\left({\mathrm{Seq}}_{\mathrm{infer}}^{F}\right)=\left(\frac{v\cdot w_{1}}{v\cdot \sum_{i=1}^{m}w_{i}},\;\ldots ,\;\frac{v\cdot w_{m}}{v\cdot \sum_{i=1}^{m}w_{i}}\right)=\left(\frac{w_{1}}{\sum_{i=1}^{m}w_{i}},\;\ldots ,\;\frac{w_{m}}{\sum_{i=1}^{m}w_{i}}\right).$$

As a note, the evaluation metric is designed for smooth travel. Certainly, some travelers possibly encounter traffic jams, red light stops or bus boarding on their journeys, which cause some base stations to have unreasonable evaluations of CMD. Nonetheless, the errors can be adaptively eliminated by our segment-grained fingerprinting, which is further demonstrated in Section 5.

Suppose $Path_{A}$ consists of a series of segments $\langle E_{1},\;E_{2},\ldots ,E_{\omega }\rangle$. $S\left(Path_{A}\right)\;$represents the SBS set derived from the pre-calibrated fingerprint map, and the number of included base stations is denoted by φ, which is

(20) $$S\left(Path_{A}\right)=S\left(E_{1},\;E_{2},\ldots ,E_{\omega }\right)=\left\{{\mathrm{BS}}_{1}{,\;\mathrm{BS}}_{2}{,\;\ldots ,\;\mathrm{BS}}_{\varphi }\right\}.$$

The candidate path selection criterion is shown as follows. For instance, the matching degree of candidate $Path_{A}$ and $Seq_{infer}^{F}$ is evaluated by Jensen–Shannon divergence [53].

(21) $$\mathrm{JS}\left(p||q\right)=\frac{1}{2}\mathrm{KL}\left(p||\frac{p+q}{2}\right)+\frac{1}{2}\mathrm{KL}\left(q||\frac{p+q}{2}\right),\;$$

(22) $$\mathrm{Similarity}\left({\mathrm{Path}}_{A}\right)=1\;-\;\mathrm{JS}\left(p||q\right).\;$$

The p denotes the proportional distribution of base station’s connection range obtained from cellular signaling sequence, which is

(23) $$p=R\left({\mathrm{Seq}}_{\mathrm{infer}}^{F}\right).$$

The q is the distribution that is constructed for the similarity comparison with p based on the pre-calibrated fingerprint map. The construction procedure is illustrated by Algorithm 2.
**Algorithm 2.** The construction of q.**Input:**$B\left({\mathrm{Seq}}_{\mathrm{infer}}^{F}\right)=\left\{{\mathrm{BS}}_{1}{,\;\mathrm{BS}}_{2}{,\;\ldots ,\;\mathrm{BS}}_{m}\right\}$$R\left({\mathrm{Seq}}_{\mathrm{infer}}^{F}\right)=\left(R_{1}{,\;R}_{2}{,\;\ldots ,\;R}_{m}\right)$$S\left({\mathrm{Path}}_{A}\right)=\left\{{\mathrm{BS}}_{1}{,\;\mathrm{BS}}_{2}{,\;\ldots ,\;\mathrm{BS}}_{\varphi }\right\}$$R\left({\mathrm{Path}}_{A}\right)=\left(R_{1}{,\;R}_{2}{,\;\ldots ,\;R}_{\varphi }\right)$**Output**:qFOR each BS IN $B\left({\mathrm{Seq}}_{\mathrm{infer}}^{F}\right)$:    IF BS is in $S\left({\mathrm{Path}}_{A}\right)$:       Take i the index of BS in $S\left(Path_{A}\right)$;       Take j the index of BS in $B\left({\mathrm{Seq}}_{\mathrm{infer}}^{F}\right)\;$;       $\;r_{j}=R_{i}$;    ELSE:       $r_{j}=0$
. ; $q=\left(\frac{r_{1}}{\sum_{j=1}^{\varphi }r_{j}},\frac{r_{2}}{\sum_{j=1}^{\varphi }r_{j}},\ldots ,\;\frac{r_{\varphi }}{\sum_{j=1}^{\varphi }r_{j}}\right)$. 

**Figure 6:**
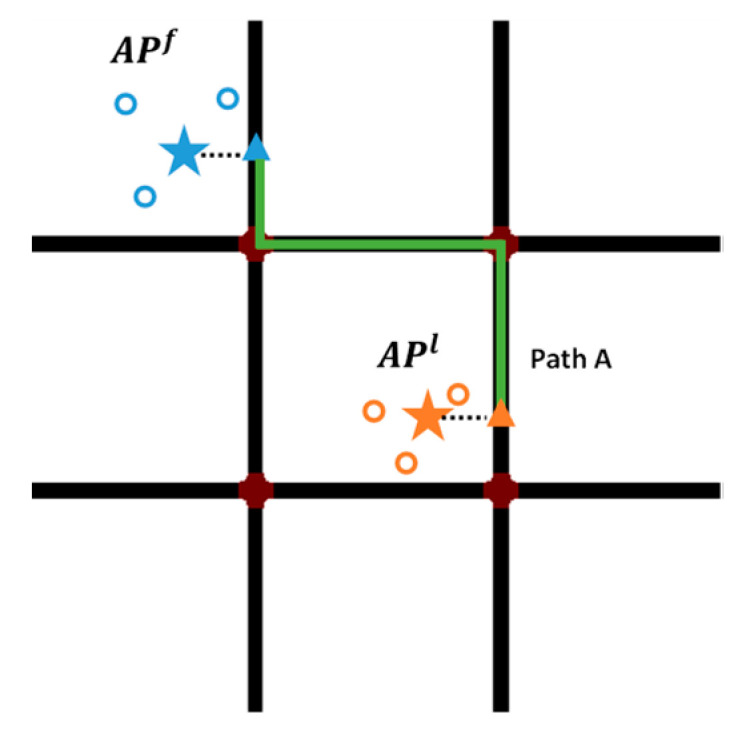
The orange anchor point is matched to the road network, and a path is reconstructed.

Finally, we compare the matching degree of $Seq_{infer}^{F}$ and candidate paths and choose the largest one as the maximum likelihood matching path. For instance, if $\mathrm{Similarity}\left(Path_{A}\right)$ is larger than $\mathrm{Similarity}\left(Path_{B}\right)$, the anchor point is matched to the nearest position of $Path_{A},$ marked as the orange triangle in Figure 6. Simultaneously, a sub-trajectory that connects the two consecutive APs is generated.

#### 4.2.3. Step 3: Trajectory Reconstruction

**Figure 7:**
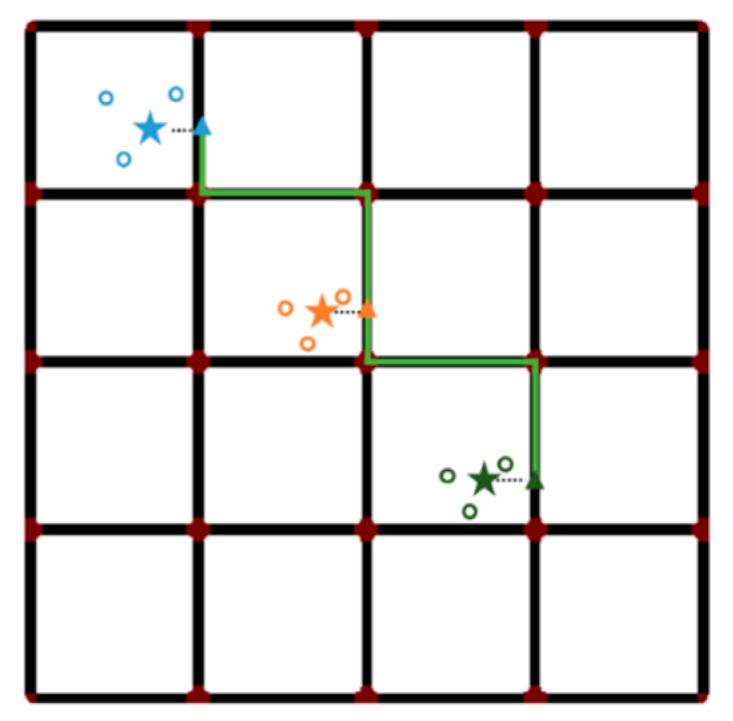
Reconstruct trajectory by connecting all matched anchor points.

After the series of anchor points is matched to the road network, the generated sub-trajectories are concatenated simultaneously to form the shortest path shown in Figure 7, which is the update of mobile device’s estimated trajectory. The road segments that the trace gets passed in the digital map are extracted and chronologically ordered. Therefore, the trajectory can be transformed into an orderly sequence of road segments $\langle E_{1},\;E_{2},\;\ldots ,\;E_{n}\rangle$, which are jointed by intersections or road junctions.

## 5. Results and Discussion

**Figure 8:**
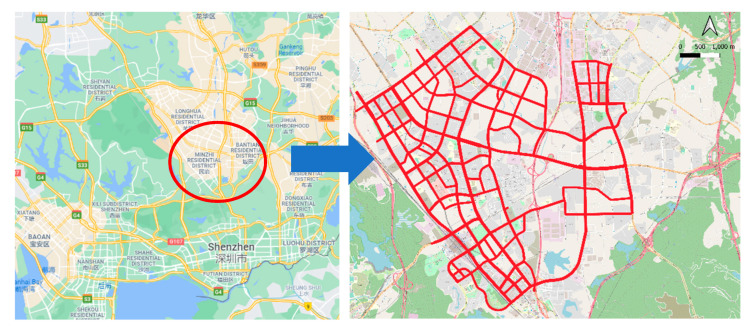
Testing area.

We conduct our experiments on Minzhi and Bantian, two adjacent subdistricts in Shenzhen, China, where there is 209.9 km of urban roads in an area of 49.7 km^2^ (Figure 8). The whole network is divided into 603 segments, which are basic fingerprint units of the fingerprint map.

Our dataset is classified into two categories: a training set and a testing set. The former is used for fingerprint map construction and the latter in online trajectory estimation scenarios.

The training set and the testing set contain 172 and 306 trajectories, respectively. We implemented the system using Python with an 8 GB RAM, 2.30 GHz core i5 processor.

### 5.1. Fingerprint Map Robustness Evaluation

To assess the robustness of the fingerprint map, we studied 24 segments with an average length of 438 m, of which each has eight cellular signaling sequences. The following two parts are robustness evaluations from the perspective of the SBS set and the impact strength of SBS, respectively.

#### 5.1.1. Part1: SBSS Evaluation

In the first part, we investigate the variation of SBSS with different numbers of the cellular signaling sequences. Let $S^{\left(k\right)}\;$be the SBSS extracted from k(1≤k≤8) sequences. For instance, $S^{\left(3\right)}\;$represents the SBSS calibrated by the three cellular signaling sequences. The variation of $S^{\left(k\right)}\;$can be measured by the Jaccard index, which is

(24) $$J\left(S^{\left(k\right)},\;S^{\left(8\right)}\right)=\frac{\left|S^{\left(k\right)}\cap \;S^{\left(8\right)}\right|}{\left|S^{\left(k\right)}\cup \;S^{\left(8\right)}\right|},$$

and note that $S^{\left(8\right)}\;$is regarded as the baseline in our case.

The variation of SBSS can be quantified by the average $J\left(S^{\left(k\right)},\;S^{\left(8\right)}\right)\;$of N (here, N=24) segments, and it is denoted by $Similarity^{\left(k\right)}$ as follows.

(25) $$Similarity^{\left(k\right)}=\frac{1}{N}\sum^{\;}J\left(S^{\left(k\right)},\;S^{\left(8\right)}\right).$$

**Figure 9:**
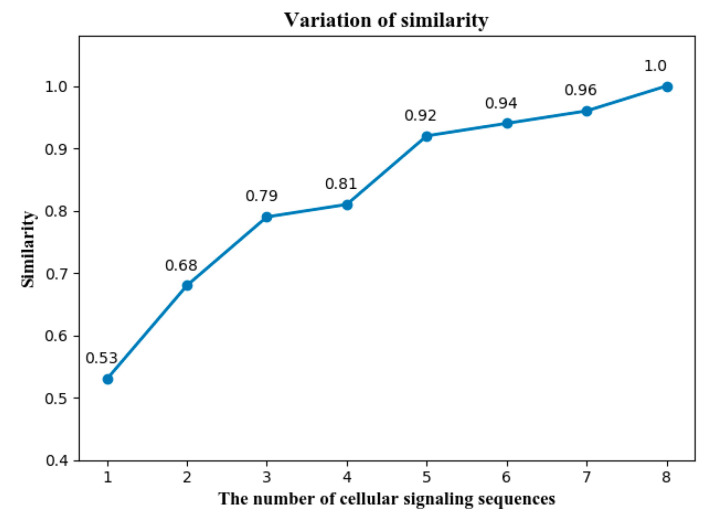
The variation of SBSS under different numbers of cellular signaling sequences.

Figure 9 shows the variation of $Similarity^{\left(k\right)},$ while the number of cellular sequences used for fingerprint calibration varies from one to eight.

It can be found that the majority of SBSs can be separated from three cellular signaling sequences, because the similarity with baseline is close to 0.8, while most SBSs can be obtained from five, because the similarity reaches up to more than 0.9.

**Figure 10:**
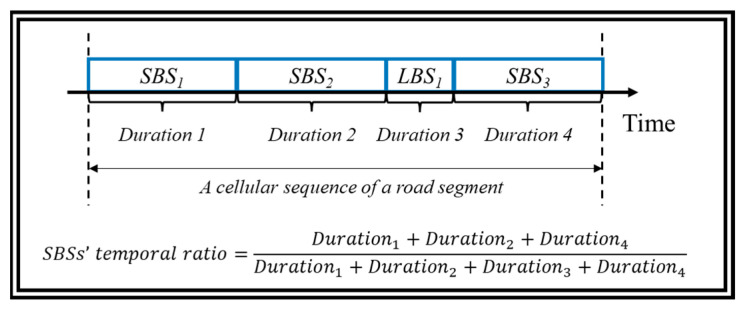
An intuitional illustration of SBSs’ temporal ratio. LBS represents the Labile impacting Base Station, which is opposite to the SBS.

The influence of SBS on each cellular signaling sequence is further inspected by SBSs’ temporal ratio, as illustrated in Figure 10 below.

**Figure 11:**
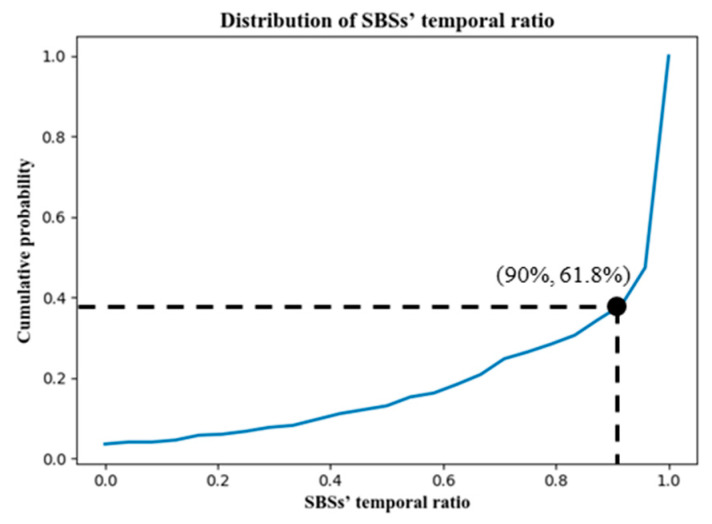
The cumulative distribution function (CDF) of SBSs’ temporal ratio.

Notice that the average SBS temporal ratio of 24 × 8 = 192 cellular signaling sequences is 83.34%. Among all cellular signaling sequences, such a ratio is up to 90% for 61.80% of them, which is shown in Figure 11.

In conclusion, the SBSs are not only the base stations with high-frequency connections but are also the dominant base stations in terms of connection duration. Therefore, it is reasonable to designate them as SBSs.

#### 5.1.2. Part2: SBSs’ Impacting Weights Evaluation

In the second part, we study the stability of the SBSs’ influence on the segments by analyzing the proportional distribution of SBSs’ impacting weights with different numbers of cellular signaling sequences.

Let $R^{\left(k\right)}\left(E_{i}\right)$ denote the proportional distribution of SBSs’ impacting weights, where $E_{i}$ is the ith road segment and k (1≤k≤8) is the number of cellular signaling sequences. Then, a weighted vector of N segments can be represented as

(26) $$R^{\left(k\right)}=\left(R^{\left(k\right)}\left(E_{1}\right){,\;R}^{\left(k\right)}\left(E_{2}\right){,\;\ldots ,\;R}^{\left(k\right)}\left(E_{N}\right)\right).\;$$

Without loss of generality, we take the value of k to be 3, 5 and 8.

**Figure 12:**
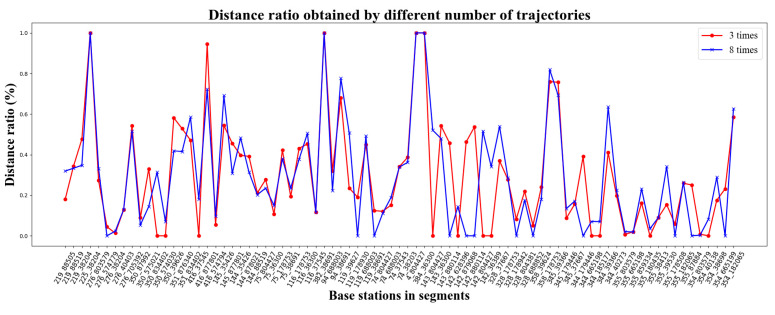
The visualization of $R^{\left(3\right)}$ and $R^{\left(8\right)}$.

Let k be equal to 3 and 8. We evaluate the similarity between $R^{\left(3\right)}$ and $R^{\left(8\right)}$ by their JS divergence, denoted by $\mathrm{JS}\left(R^{\left(3\right)}||R^{\left(8\right)}\right)$, which is formulated as Equation (21). The value of $\mathrm{JS}\left(R^{\left(3\right)}||R^{\left(8\right)}\right)$ is 0.08. That means there is a certain resemblance between $R^{\left(3\right)}$ and $R^{\left(8\right)}$. To show the similarity intuitively, Figure 12 plots these two impacting weights of the SBSs.

**Figure 13:**
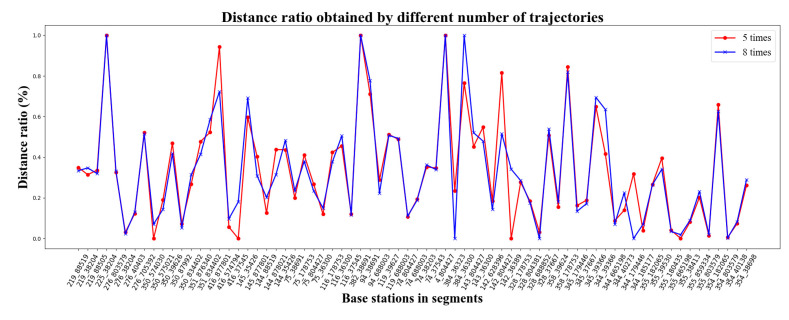
The visualization of $R^{\left(5\right)}$ and $R^{\left(8\right)}$.

Likewise, we can evaluate the similarity between $R^{\left(5\right)}$ and $R^{\left(8\right)}$ by $\mathrm{JS}\left(R^{\left(5\right)}||R^{\left(8\right)}\right)$, whose value is further down to 0.02. $R^{\left(5\right)}$ and $R^{\left(8\right)}$ are comparatively shown in Figure 13. It can be found that the proportionality of SBSs’ weights obtained from five cellular sequences is highly identical to that from eight cellular sequences.

In summary, the major fingerprint features of the road segment can be extracted from three cellular signaling sequences, and five cellular signaling sequences can provide extremely accurate fingerprint features. In our case, each road segment has no less than five cellular sequences for fingerprint feature calibration.

### 5.2. Model Performance

In the experimental region, we tested 306 cellular signaling sequences whose corresponding trajectories varied from 1 km to 6 km in length, and their total was 1367 km.

#### 5.2.1. Metrics

The performance of trajectory estimation algorithm is evaluated by the precision rate and the recall rate as below.

(27) $$\mathrm{Precision}=\frac{\left|\;X\;\cap \;Y\;\right|}{\left|\;X\;\right|},\;$$

(28) $$\mathrm{Recall}=\frac{\left|\;X\;\cap \;Y\;\right|}{\left|Y\right|}\;$$

**Figure 14:**
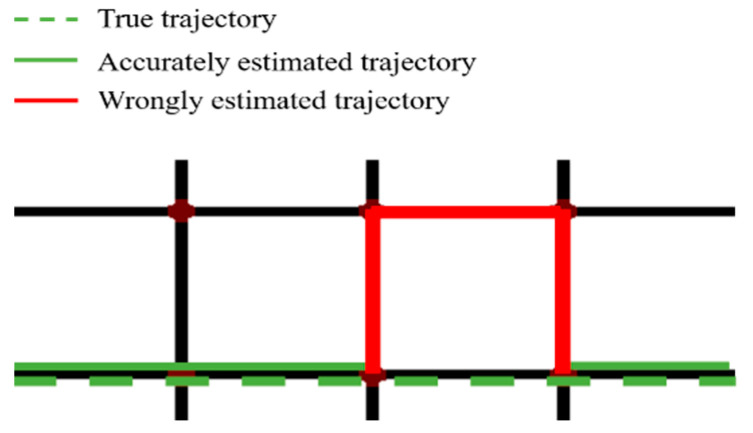
An illustration for the performance of trajectory estimation.

Herein, X represents the estimated trajectory (the solid line in Figure 14), Y represents the true trajectory (the green dash line) and $X\cap^{\;}Y$ represents the overlapping part of the two trajectories (the green solid line), namely the correct estimation. Their lengths are denoted by |X|, |Y| and $\left|X\cap^{\;}Y\right|$ respectively.

#### 5.2.2. Parametric Studies

The systematic performance of our model is affected by many parameters. In this chapter, the critical parameters of NF-Track are further investigated as follows.

(1) The θ-value of segment fingerprinting

**Figure 15:**
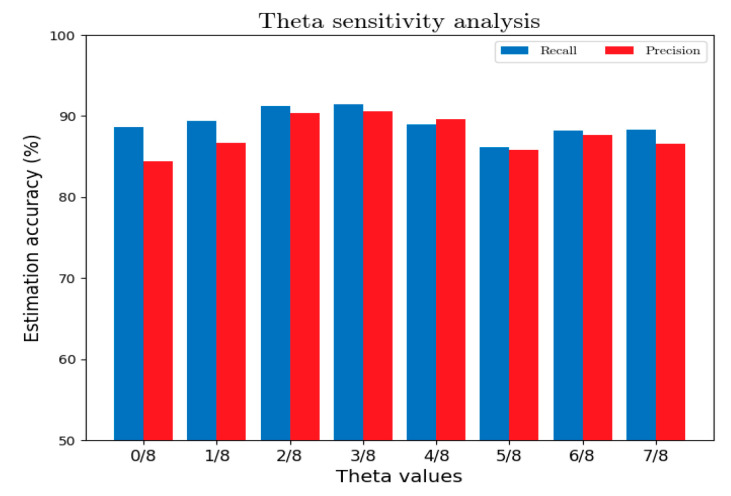
Effects of the θ-value on the fingerprinting performance.

A small θ-value results in the underfitting of segment fingerprinting, while a large one leads to overfitting. Therefore, the effect of changing the θ-value on the overall accuracy was investigated in this study at a 180-s time span, which proved later that the algorithm can reach the optimum. Figure 15 shows that, with increasing the θ-value, the accuracy increases at first and then decreases. Hence, θ-value was set to 0.375 optimally, as shown in Figure 15.

(2) The ε-value of anchor clustering

**Figure 16:**
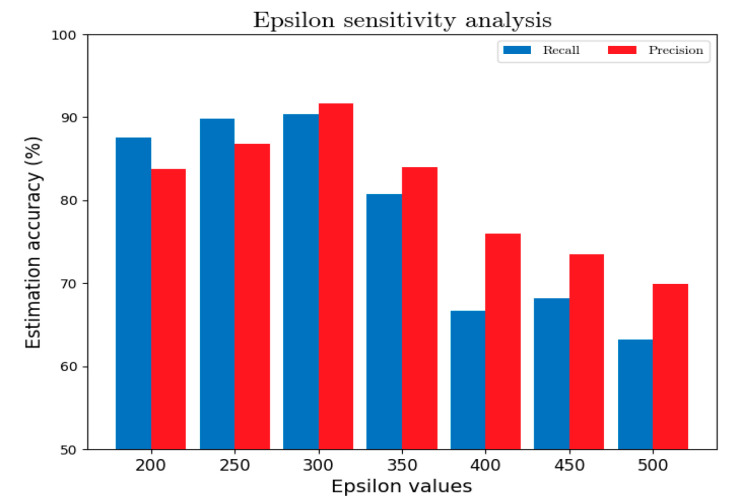
Effects of the ε-value on the fingerprinting performance.

The number of APs obtained from the cellular trajectory is mainly affected by the ε-neighborhood in the density-based clustering algorithm. More APs bring more locating information to the real-time tracking, but more noise is introduced at the same time. Thus, it needs some tuning to fetch a suitable ε-value. Figure 16 shows the effect of changing the ε-value in the estimation accuracy. It can be seen that the system achieves the best performance at ε=300 within the range of testing.

(3) The searching radius of anchor map matching

**Figure 17:**
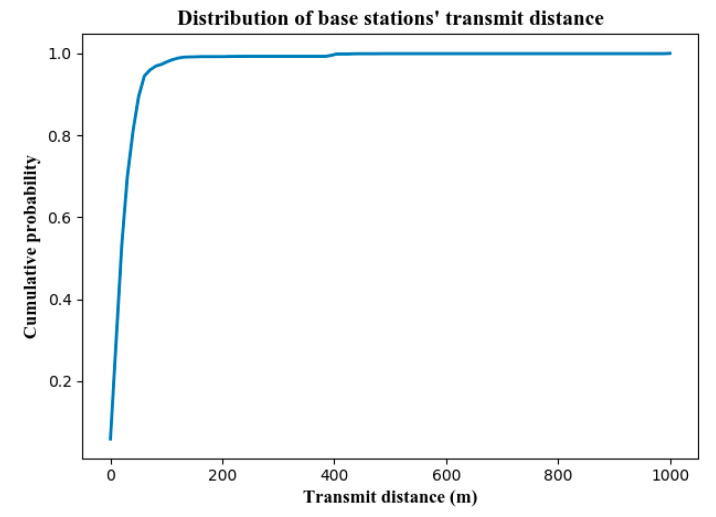
The distribution of transmitting distance between base stations and mobile devices in our testing experiment.

The searching radius of anchor map matching mentioned in Section 4.2.2 was set to 800 m here, because the result of a testing experiment shown in Figure 17 indicated that the transmit distance of most base stations does not exceed 800 m. Hence, such a searching radius ensures the accurate map-matching of anchor points on ordinary urban road networks. The anchor was discarded when it could not find any segment within 800 m.

#### 5.2.3. Overall System Performance

NF-Track is compared with three types of RSS-independent trajectory estimation algorithms, including (a) SnapNet [26]: The current state-of-the-art online cellular trajectory estimation algorithm in map-matching cellular-based locations with several innovative filters. (b) standardHMM: A plain HMM technique that map-matches the cellular locations without using any of the additional filters. (c) NearRd: Map-matching the anchor points to the road nearby and connecting them through the shortest path without the fingerprint map.

**Figure 18:**
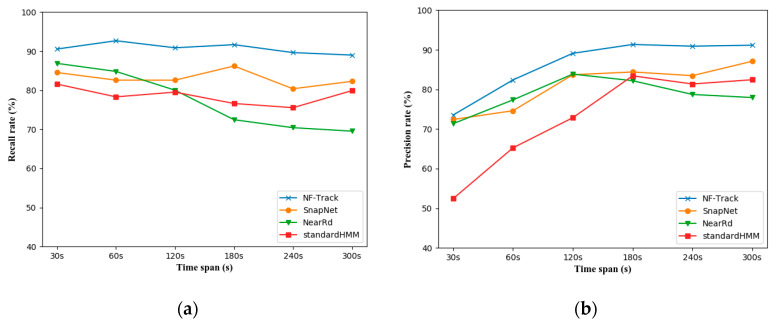
Comparison of map-matching accuracy on the real dataset for various update spans. (**a**) Recall rates and (**b**) precision rates.

Figure 18a,b shows their recall rates and precision rates to evaluate the accuracy for various trajectory update spans w. It can be seen that all algorithms are difficult to obtain high accuracy when the update span is less than 60 s due to the high noise and sparseness of cellular-based positioning. NF-Track has extremely higher accuracy than other state-of-the-art online map-matching algorithms in all update spans and performs the best with 91.68% recall and 90.35% precision at w=180 s. If we perform map-matching without a fingerprint map (NearRd), the accuracies have a general decline of more than ten percent, which suggests that the fingerprint map is able to enhance the effect of the trajectory estimation greatly.

**Figure 19:**
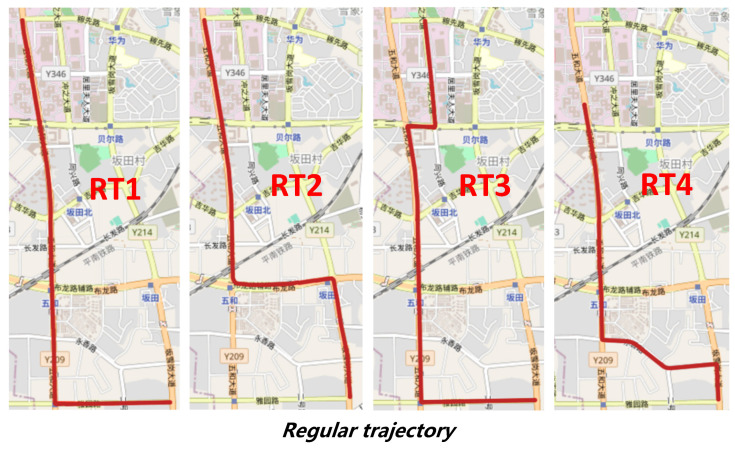

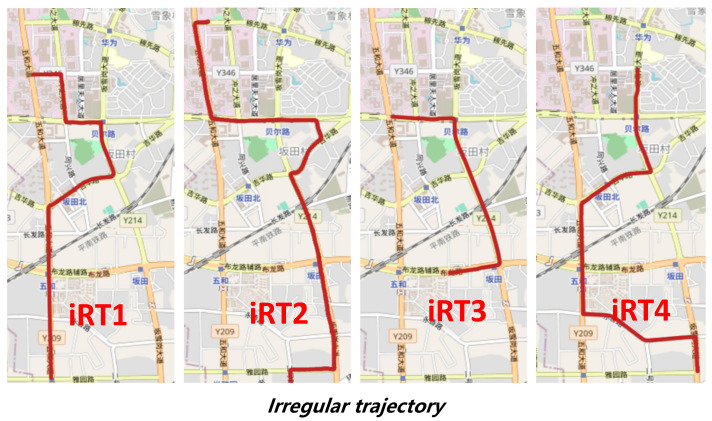
Regular trajectory (RT) and irregular trajectory (iRT).

To illustrate the advantage over the current state-of-the-art unsupervised methods, we further compare NF-Track with SnapNet in two categories of trajectory: regular trajectory (RT) and irregular trajectory (iRT), shown in Figure 19. The regular trajectories are generated by travelers that follow the shortest and straightest paths. Besides, some trajectories in our testing dataset are longer and more twisted because of many irregular circumstances, e.g., congestion avoidance and bus detouring, and we call them irregular trajectories.

**Figure 20:**
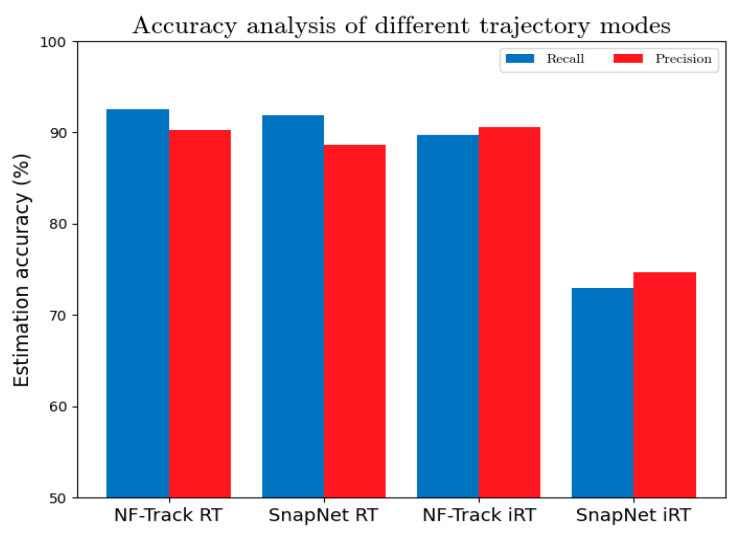
The estimation accuracy of NF-Track and SnapNet on RT and iRT.

Figure 20 shows two algorithms’ estimation accuracy on two kinds of trajectories at w=180 s, where both supervised and unsupervised models achieve satisfactory performances, as seen in Figure 20. It follows that both models acquire satisfactory results for regular trajectories. However, for irregular trajectories, NF-Track is obviously more effective, while unsupervised models are not robust, which makes it unsuitable for map-matching in complicated urban environments. Moreover, NF-Track has low sensitivity to the patterns of moving trajectory, which indicates that the fingerprinting mechanism is helpful for enhancing the stability and generalization of cellular trajectory map-matching.

### 5.3. Algorithm Evaluation

AP clustering and its timestamp inference are vital to the trajectory estimation algorithm. The correctness of AP map-matching is mainly affected by the inferred timestamps of its previous AP. Hence, it is essential to evaluate the accuracy of inferred timestamps and their influence on the map-matching, so as to assess the validity of the trajectory estimation algorithm.

In this study, 976 APs are extracted for analysis from the 306 cellular signaling sequences at w=180 s. For each cellular signaling sequence, its extracted AP series is the backbone to reconstruct the spatiotemporal trajectory of the mobile device. As mentioned above, each GPS location recorded has already been matched to a certain position in the road network. Therefore, as for an AP, its closest GPS point can be taken as the true position where it should be matched. The timestamp of such a GPS location point, which is denoted by $T_{true}$, is regarded as the true value of AP’s timestamp.

Referring to its inferred timestamp $T_{infer}$ derived by Equations (13) and (14), the timestamp inferring error of AP is expressed as:

(29) $$\mathrm{Error}\left(\mathrm{AP}\right)=\left|T_{\mathrm{true}}{-T}_{\mathrm{infer}}\right|.$$

**Figure 21:**
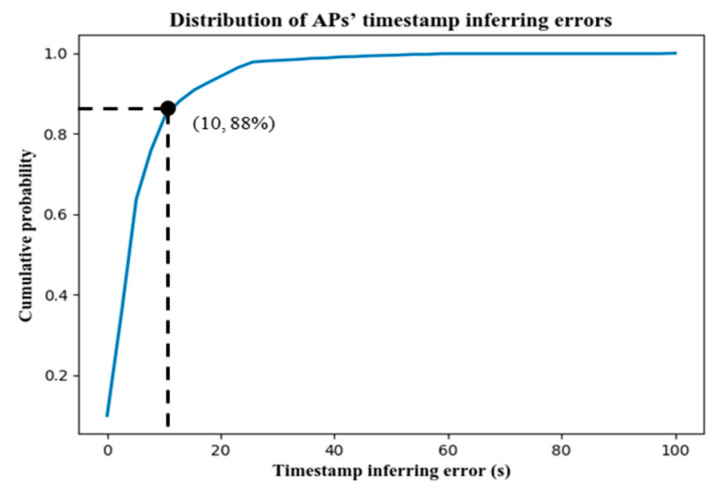
The CDF of AP’s timestamp inferring errors.

Through the analysis of these 976 APs, the distribution of their timestamp inferring error is obtained as Figure 21. Herein, 88% of the anchor points have errors within 10 s. The large inferring errors of a small part of the APs are caused by the devices’ long rest behavior mentioned above, which brings perturbation to the timestamp inference. Overall, from the error distribution of APs’ timestamps inference, such perturbations brought by the unsteady movement of urban travelers can be tolerated, because the average time gap of two successive APs is up to 93 s.

To further investigate the influence of timestamp inferring errors on fingerprint comparison and map-matching of APs, the cellular signaling fragments truncated by two consecutive APs are explored.

Suppose $AP^{f}\;$and $AP^{l}\;$are two successive anchor points. The inferred timestamp and true timestamp of $AP^{f}$ are $T_{infer}^{f}$ and $T_{true}^{f}$. $T_{infer}^{l}$ and $T_{true}^{l}$ are corresponding timestamps of $AP^{l}$. $Seq_{infer}^{F}$ and $Seq_{true}^{F}$ are cellular signaling fragments of time intervals $\left[T_{infer}^{f},\;T_{infer}^{l}\right]$ and $[T_{true}^{f},\;T_{true}^{l}],$ respectively.

$B\left(Seq_{infer}^{F}\right)\;$represents the base station set that consists of all the recorded base stations in $Seq_{infer}^{F}$. Likewise, $B\left(Seq_{true}^{F}\right)\;$is derived from $Seq_{true}^{F}$. The consistency of $B\left(Seq_{infer}^{F}\right)\;$to $B\left(Seq_{true}^{F}\right)\;$is evaluated as follows:

(30) $$\mathrm{Consistency}=\frac{\left|B\left(Seq_{infer}^{F}\right)\;\cap \;B\left(Seq_{true}^{F}\right)\right|}{\left|B\left(Seq_{true}^{F}\right)\right|},$$

For all truncated cellular signaling fragments, the average consistency is 0.78, which indicates that the errors of inferred base station sets are quite small.

**Figure 22:**
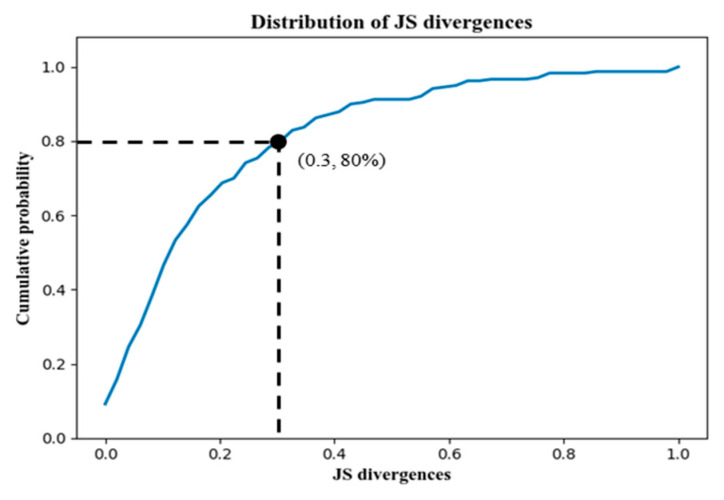
The CDF of $\mathrm{JS}\left(R\left(Seq_{infer}^{F}\right)||R\left(Seq_{true}^{F}\right)\right).$

The proportion of base stations’ CMD in $Seq_{infer}^{F}$ and $Seq_{true}^{F}$, respectively, denoted by $R\left(Seq_{infer}^{F}\right)$ and $R\left(Seq_{true}^{F}\right)$, were further evaluated by JS divergence $\mathrm{JS}\left(R\left(Seq_{infer}^{F}\right)||R\left(Seq_{true}^{F}\right)\right)$ to observe their consistency. We could find that the average divergence was 0.13, and 80% of the cellular signaling fragments had a divergence less than 0.3, as shown in Figure 22.

In general, the current inferring errors of APs have a small influence on AP map-matching and trajectory estimation.

### 5.4. Further Investigation

#### 5.4.1. External Influences

Cellular signals are affected by time periods and weather conditions, to some extent. The urban traffic changes over time, which greatly influence the network load and radio signal quality [54]. Besides, outdoor weather conditions can cause severe degradation in network performance [55]. Thus, the influences of these external factors on the estimation accuracy are further investigated in this study. Table 3 summarizes the results at w=180 s. It can be seen that the overall estimation accuracy of the cellular trajectories during non-rush hours is slightly higher in contrast to the rush hours (7–10 a.m. and 5–7 p.m.). The main reason is that the cellular network has sufficient capacity, and the localization is less disturbed by the “Ping-Pong effect” during non-rush hours. Referring to historical weather information [56], we found that weather conditions have little impact on the performance of NF-Track, because dense base stations are deployed in urban areas, and they serve mobile users well.

#### 5.4.2. Computing Latency

**Figure 23:**
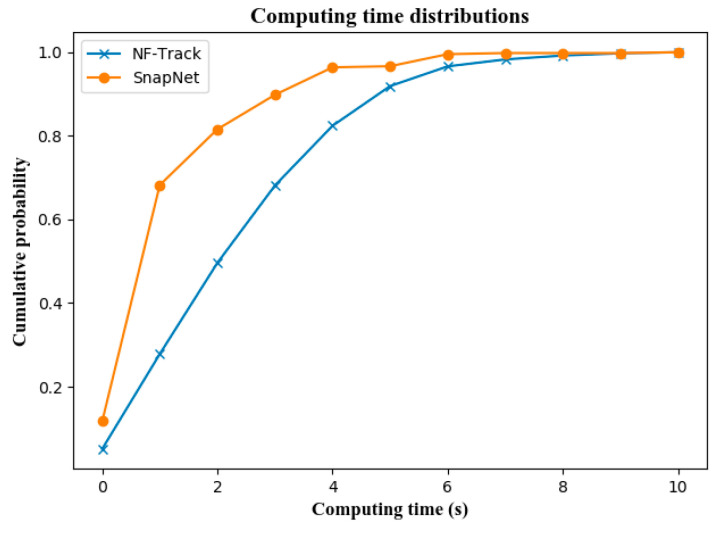
The computing latency per trajectory update.

The computing consumption of several online trajectory estimation methods mentioned above is further investigated. Figure 23 presents the computational efficiency of NF-Track and SnapNet, respectively, which is the computing latency to process a timespan of input cellular locations. It can be seen that, although the computation of NF-Track is a bit longer than the unsupervised method due to the fine-grained fingerprint comparison implemented in dense urban road networks, it is worthy of significant improvement in the estimation accuracy.

## 6. Conclusions

Cellular signaling data contains wealthy movement information of urban travelers. In this paper, we proposed NF-Track to realize the online trajectory estimations in urban road networks based on a network-wide fingerprint map. Different from previous cellular signaling sequence map-matching systems, NF-Track is independent of either hardware-relevant information in conventional fingerprinting approaches or heuristic hypotheses that are widely leveraged by unsupervised methods. Therefore, NF-Track is more suitable for cloud computing backend deployment to realize real-time tracking, which benefits many applications, such as infectious disease tracing and screening, network flow sensing and traffic scheduling.

NF-Track is tested on a real-world urban dataset. The experiment shows that our novel fingerprint map is robust enough to assist in the accurate map-matching of cellular signaling sequences. NF-Track can achieve a recall rate of 91.68% and a precision rate of 90.35% in sophisticated traffic scenes, which are superior to the state-of-the-art model-based unsupervised learning approaches.

NF-Track has great potential to be extended over larger areas. For this purpose, we are currently making efforts to enhance the generalization and portability of NF-Track. In particular, we have involved the base station parameters in the fingerprinting process, which can further facilitate the deployment of our systems and maintain the estimation accuracy in a larger urban area.

## Figures and Tables

**Table 1:** Information of the acquired data.

Device ID	Timestamp	Base Station ID	GPS Longitude	GPS Latitude
199**19	2020-10-10 09:06:48	688**3	22.620568	114.055819
199**19	2020-10-10 09:06:49	688**3	22.620752	114.055815
199**19	2020-10-10 09:06:50	181**8	22.620910	114.055811

**Table 2:** Main notations in this paper.

Seq	Cellular signaling sequence	$T_{true}$	True timestamp of AP
E	Basic road segment for fingerprinting	$T_{infer}$	Inferred timestamp of AP
BS	Base station	$AP^{f}$	The former one of two successive anchor points
SBS	Stable impacting base station	$AP^{l}$	The latter one of two successive anchor points
SBSS	Stable impacting base station set	$T_{infer}^{f}$	Inferred timestamp of $AP^{f}$
S(E)	SBSS of E	$T_{true}^{f}$	True timestamp of $AP^{f}$
CMD	Cumulative moving distance	$T_{infer}^{l}$	Inferred timestamp of $AP^{l}$
$\bar{CMD}$	Average CMD obtained from several cellular sequences	$T_{true}^{l}$	True timestamp of $AP^{l}$
R	Impacting intensity of an SBS	$Seq_{infer}^{F}$	Cellular signaling fragment between $\left(T_{infer}^{f},\;T_{infer}^{l}\;\right)$
R(E)	Fingerprint of E	$Seq_{true}^{F}$	Cellular signaling fragment between $\left(T_{true}^{f},\;T_{true}^{l}\;\right)$
P	Path that consists of a segment series	$B\left(Seq_{infer}^{F}\right)$	Base station set of $Seq_{infer}^{F}$
S(P)	Integrated SBSS of P	$R\left(Seq_{infer}^{F}\right)$	Estimated CMD proportion of $B\left(Seq_{infer}^{F}\right)$
R(P)	Integrated fingerprint of P	$B\left(Seq_{true}^{F}\right)$	Base station set of $Seq_{true}^{F}$
AP	Anchor point	$R\left(Seq_{true}^{F}\right)$	Estimated CMD proportion of $B\left(Seq_{true}^{F}\right)$

**Table 3:** The influence of external factors on the estimation accuracy.

Influencing Factors		Recall	Precision
Time period	Rush	92.26%	88.38%
	Non-Rush	91.43%	91.20%
Weather condition	Sunny	92.29%	90.59%
	Cloudy	91.19%	90.15%
	Rainy	91.99%	90.48%

## References

[1] Du R., Santi P., Xiao M., Vasilakos A.V., Fischione C. (2018). The sensable city: A survey on the deployment and management for smart city monitoring. IEEE Commun. Surv. Tutor..

[2] Ren Y., Wang T., Zhang S., Zhang J. (2020). An intelligent big data collection technology based on micro mobile data centers for crowdsensing vehicular sensor network. Pers. Ubiquitous Comput..

[3] Ghahramani M., Zhou M.C., Wang G. (2020). Urban sensing based on mobile phone data: Approaches, applications, and challenges. IEEE-CAA J. Autom. Sin..

[4] Goh C.Y., Dauwels J., Mitrovic N., Asif M.T. Online map-matching based on hidden markov model for real-time traffic sensing applications. Proceedings of the 2012 15th International IEEE Conference on Intelligent Transportation Systems (ITSC).

[5] Lu Y., Ding H., Ji S., Sze N.N., He Z. (2021). Dual attentive graph neural network for metro passenger flow prediction. Neural Comput. Appl..

[6] Lu Y., He Z., Luo L. Learning trajectories as words: A probabilistic generative model for destination prediction. Proceedings of the 16th EAI International Conference on Mobile and Ubiquitous Systems: Computing, Networking and Services.

[7] Michael W.J., Leo S., David G.S. (1999). Combining GPS with TOA/TDOA of Cellular Signals to Locate Terminal. U.S. Patent.

[8] Ibrahim M., Youssef M. (2012). CellSense: An Accurate Energy-Efficient GSM Positioning System. IEEE Trans. Veh. Technol..

[9] Wymeersch H., Seco-Granados G., Destino G., Dardari D., Tufvesson F. (2017). 5G mmWave Positioning for Vehicular Networks. IEEE Wirel. Commun..

[10] Laoudias C., Moreira A., Kim S., Lee S., Wirola L., Fischione C. (2018). A Survey of Enabling Technologies for Network Localization, Tracking, and Navigation. IEEE Commun. Surv. Tutor..

[11] Xiao Z., Zeng Y. (2022). An Overview on Integrated Localization and Communication towards 6G. Sci. China Inf. Sci..

[12] Servick K. (2020). Cellphone tracking could help stem the spread of coronavirus. Is privacy the price?. Science.

[13] Rosenkrantz L., Schuurman N., Bell N., Amram O. (2020). The need for GIScience in mapping COVID-19. Health Place.

[14] Sun H.-C., Liu X.-F., Du Z.-W., Xu X.-K., Wu Y. (2021). Mitigating COVID-19 Transmission in Schools with Digital Contact Tracing. IEEE Trans. Comput. Soc. Syst..

[15] Liu Z., Liu Y., Meng Q., Cheng Q. (2019). A tailored machine learning approach for urban transport network flow estimation. Transp. Res. Part C Emerg. Technol..

[16] Wu C., Thai J., Yadlowsky S., Pozdnoukhov A., Bayen A. (2015). Cellpath: Fusion of cellular and traffic sensor data for route flow estimation via convex optimization. Transp. Res. Part C Emerg. Technol..

[17] Breyer N., Gundlegård D., Rydergren C. (2021). Travel mode classification of intercity trips using cellular network data. Transp. Res. Procedia..

[18] Liu T., Wang F.-Y., Tian B., Ai Y., Chen L. (2019). Parallel distance: A new paradigm of measurement for parallel driving. IEEE/CAA J. Autom. Sin..

[19] Zhang T., Song W., Fu M., Yang Y., Wang M. (2021). Vehicle motion prediction at intersections based on the turning intention and prior trajectories model. IEEE/CAA J. Autom. Sin..

[20] Gao K., Zhang Y., Su R., Yang F., Suganthan P., Zhou M. (2019). Solving Traffic Signal Scheduling Problems in Heterogeneous Traffic Network by Using Meta-Heuristics. IEEE Trans. Intell. Transp. Syst..

[21] Hoteit S., Secci S., Sobolevsky S., Pujolle G., Ratti C. Estimating real human trajectories through mobile phone data. Proceedings of the 2013 IEEE 14th International Conference on Mobile Data Management.

[22] Li M., Gao S., Lu F., Zhang H. (2019). Reconstruction of human movement trajectories from large-scale low-frequency mobile phone data. Comput. Environ. Urban Syst..

[23] Schulze G., Horn G., Kern R. Map-matching cell phone trajectories of low spatial and temporal accuracy. Proceedings of the 2015 IEEE 18th International Conference on Intelligent Transportation Systems.

[24] Bonnetain L., Furno A., El Faouzi N.-E., Fiore M., Stanica R., Smoreda Z., Ziemlicki C. (2021). TRANSIT: Fine-grained human mobility trajectory inference at scale with mobile network signaling data. Transp. Res. Part C Emerg. Technol..

[25] Shen Z., Du W., Zhao X., Zou J. DMM: Fast map matching for cellular data. Proceedings of the 26th Annual International Conference on Mobile Computing and Networking.

[26] Mohamed R., Aly H., Youssef M. (2017). Accurate Real-time Map Matching for Challenging Environments. IEEE Trans. Intell. Transp. Syst..

[27] Jagadeesh G.R., Srikanthan T. (2017). Online map-matching of noisy and sparse location data with hidden Markov and route choice models. IEEE Trans. Intell. Transp. Syst..

[28] Bonnetain L., Furno A., Krug J., El Faouzi N.-E. (2019). Can We Map-Match Individual Cellular Network Signaling Trajectories in Urban Environments? Data-Driven Study. Transp. Res. Rec. J. Transp. Res. Board..

[29] Bahl P., Padmanabhan V. RADAR: An In-building RF-based User Location and Tracking System. Proceedings of the IEEE Infocom, Tel-Aviv.

[30] Kaemarungsi K., Krishnamurthy P. Modeling of indoor positioning systems based on location fingerprinting. Proceedings of the IEEE INFOCOM 2004–The Conference on Computer Communications–Twenty Third Annual Joint Conference of the IEEE Computer and Communications Societies.

[31] Shin H., Cha H. Wi-Fi Fingerprint-Based Topological Map Building for Indoor User Tracking. Proceedings of the 16th IEEE International Conference on Embedded and Real-Time Computing Systems and Applications, RTCSA’10.

[32] Li Q., Liao X., Liu M., Valaee S. (2021). Indoor Localization Based on CSI Fingerprint by Siamese Convolution Neural Network. IEEE Trans. Veh. Technol..

[33] Ergen S.C., Tetikol H.S., Kontik M., Sevlian R., Rajagopal R., Varaiya P. (2014). RSSI-fingerprinting-based mobile phone localization with route constraints. IEEE Trans. Veh. Technol..

[34] Thiagarajan A., Ravindranath L.S., Balakrishnan H., Madden S., Girod L. Accurate, Low-Energy Trajectory Mapping for Mobile Devices. Proceedings of the 8th USENIX Symposium on Networked Systems Design and Implementation (NSDI 2011).

[35] Torre A.D., Gallo P., Gubiani D., Marshall C., Montanari A., Pittino F. (2019). A map-matching algorithm dealing with sparse cellular fingerprint observations. Geo-Spatial Inf. Sci..

[36] Gundlegård D., Karlsson J.M. (2020). Integrated tracking and route classification for travel time estimation based on cellular network signalling data. IET Intell. Transp. Syst..

[37] Wu H., Sun W., Zheng B., Yang L., Zhou W. (2017). CLSTERS: A General System for Reducing Errors of Trajectories Under Challenging Localization Situations. ACM Interact. Mob. Wearable Ubiquitous Technol..

[38] Karimi H.A. (2016). Advanced Location-Based Technologies and Services.

[39] Chen M.Y., Sohn T., Chmelev D., Hähnel D. (2006). Practical metropolitan-scale positioning for gsm phones. Proceedings of the International Conference Ubiquitous Computing.

[40] Greenfeld J.S. Matching GPS observations to locations on a digital map. Proceedings of the Transportation Research Board 81st Annual Meeting.

[41] Brakatsoulas S., Pfoser D., Salas R., Wenk C. On Map-Matching Vehicle Tracking Data. Proceedings of the 31st Inter-national Conference on Very Large Data Bases (VLDB).

[42] He Z.-C., Xi-Wei S., Nie P.-L., Zhuang L.-J. (2013). On-line map-matching framework for floating car data with low sampling rate in urban road networks. IET Intell. Transp. Syst..

[43] Lou Y., Zhang C., Zheng Y., Xie X., Wang W., Huang Y. Map-matching for low-sampling-rate GPS trajectories. Proceedings of the 17th ACM SIGSPATIAL International Conference on Advances in Geographic Information Systems GIS 09.

[44] Dubé R., Sommer H., Gawel A., Bosse M., Siegwart R. Non-uniform sampling strategies for continuous correction based trajectory estimation. Proceedings of the 2016 IEEE International Conference on Robotics and Automation (ICRA).

[45] Kamali T., Stashuk D.W. (2020). Discovering Density-Based Clustering Structures Using Neighborhood Distance Entropy Consistency. IEEE Trans. Comput. Soc. Syst..

[46] Ghahramani M., O’Hagan A., Zhou M., Sweeney J. (2021). Intelligent Geodemographic Clustering Based on Neural Network and Particle Swarm Optimization. IEEE Trans. Syst. Man Cybern..

[47] Zhang Y., Cai J. (2021). Fuzzy clustering based on automated feature pattern-driven similarity matrix reduction. IEEE Trans. Comput. Soc. Syst..

[48] Dang L., Chen B., Huang Y., Zhang Y., Zhao H. (2021). Cubature Kalman Filter Under Minimum Error Entropy With Fiducial Points for INS/GPS Integration. IEEE/CAA J. Autom. Sin..

[49] Yuan Y., Wei G., Wei Q. Map matching of mobile probes based on handover location technology. Proceedings of the IEEE International Conference on Networking, Sensing and Control.

[50] Bertini R., Lovell D. (2009). Impacts of Sensor Spacing on Accurate Freeway Travel Time Estimation for Traveler Information. J. Intell. Transp. Syst..

[51] Kim H., Kim Y., Jang K. (2017). Systematic Relation of Estimated Travel Speed and Actual Travel Speed. IEEE Trans. Intell. Transp. Syst..

[52] Ester M., Kriegel H.P., Sander J., Xu X. A density-based algorithm for discovering clusters in large spatial databases with noise. Proceedings of the 2nd International Conference Knowledge Discovery and Data Mining (KDD’96).

[53] Lin J. (1991). Divergence measures based on the Shannon entropy. IEEE Trans. Inform. Theory..

[54] Paul U., Subramanian A.P., Buddhikot M.M., Das S.R. (2011). Understanding traffic dynamics in cellular data networks. Proceedings of the 2011 Proceedings IEEE INFOCOM.

[55] Luomala J., Hakala I. Effects of Temperature and Humidity on Radio Signal Strength in Outdoor Wireless Sensor Networks. Proceedings of the 2015 Federated Conference on Computer Science and Information Systems (FedCSIS).

[56] The Historical Weather in Shenzhen, China. http://lishi.tianqi.com/shenzhen/.

